# Antiulcerative and antioxidant action of hydroalcoholic extract of *Anacardium occidentale* L. leaves in an induced experimental colitis model

**DOI:** 10.1590/acb406025

**Published:** 2025-07-18

**Authors:** Victor Pedro, Maria do Socorro Medeiros Amarante, Everlândja Gomes de Almeida, Manoel André de Souza, Silvana Maria Zucolotto, Matheus Augusto de Bittencourt Pasquali, Sérgio Adriane Bezerra de Moura, Fabiane Ferreira Martins, Christina da Silva Camillo

**Affiliations:** 1 Universidade Federal do Rio Grande do Norte – Department of Morphology – Biosciences Center – Laboratory of Histotechnics – Natal (RN) – Brazil.; 2 Universidade Federal do Rio Grande do Norte – Health Sciences Center – Department of Pharmacy – Natal (RN) – Brazil.; 3 Universidade Federal do Rio Grande do Norte – Institute of Tropical Medicine – Biosciences Center – Natal (RN), Brazil.

**Keywords:** Colitis, Anacardium, Oxidative Stress, Acetic Acid, Sulfasalazine

## Abstract

**Purpose:**

To examine the antiulcerative and antioxidant action of the *Anacardium occidentale* hydroalcoholic leaf extract (HEA) on experimentally induced colitis in rats via acetic acid (AA).

**Methods:**

Male rats were distributed into six groups (n = 10 per group): C (control), CC (colitis control), SZC (sulfasalazine 500 mg/kg), and three doses of HEA (HEA50 = 50 mg/kg, HEA100 = 100 mg/kg, HEA200 = 200 mg/kg).

**Results:**

The treatment with HEA100 for seven days decreased diarrhea, increased food intake, attenuated weight loss, and recovered the macroscopic and histological parameters of the colon, mitigating the severity of colitis and restoring the intestinal morphophysiology of animals with induced colitis. Additionally, HEA50 and HEA100 treatment increased the activity of superoxide dismutase, catalase, and thiol, and reduced the levels of thiobarbituric acid reactive substances and carbonyl, contributing to the re-establishment of antioxidant homeostasis.

**Conclusion:**

This study provided preclinical evidence of the potential of *A. occidentale* leaf extract, particularly at the concentration of 100 mg/kg, as an antiulcer agent for attenuating colitis in rats, likely due to the positive modulation of the antioxidant system.

## Introduction

Ulcerative colitis (UC) is a chronic inflammatory disease that affects the colon mucosa; its genesis is related to multiple factors, including heredity, immune deficiency, failures in the integrity of the intestinal epithelial barrier, dysbiosis, and dietary habits^1^. The clinical manifestations of UC are demarcated by abdominal pain, bloody diarrhea, fecal incontinence, and fatigue^2^. Worldwide, the occurrence of UC is increasing with annual incidences of 8.8 to 23.1 per 100,000 person-years in North America, 0.6 to 24.3 per 100,000 person-years in Europe, and 7.3 to 17.4 in Oceania, generating morbidity and economic impact on health systems^3^.

Pharmacological approaches to treating UC include 5-aminosalicylate (sulfasalazine), glucocorticoids, antibiotics, and immune modulators^4^. These pharmacological agents generate complications such as headache, diarrhea, cramps, abdominal pain, renal impairment, hyperglycemia, hypertension, and electrolyte disturbances^5^. Due to these side effects, new therapeutic proposals are necessary for the clinical management of UC.

*Anacardium occidentale*, commonly known as the cashew tree or *cajueiro* in Portuguese, is a species native to Brazil and is predominantly found in the northeastern region, especially in the state of Rio Grande do Norte^6^. Various parts of *A. occidentale* (including leaves, flowers, fruit, and pseudo fruit) have demonstrated diverse medicinal properties in preclinical studies and are widely recognized in traditional medicine. Its applications include the treatment of wounds, inflammatory conditions, infections, diabetes, diarrhea, and bleeding^7^–^9^.

The ethnopharmacological properties of *A. occidentale* are attributed to its content of alkaloids, phenolic compounds, saponins, tannins, carotenoids, and flavonoids^10^,^11^. Several preclinical studies have provided scientific evidence for the protective, anti-inflammatory, and antioxidant effects of *A. occidentale* in metabolic syndrome, neuroinflammation, diabetes, and tissue repair^12^–^14^. However, up to now, its therapeutic effects in the context of UC remain unexplored.

The experimental model of colitis is commonly induced by the intrarectal administration of acetic acid, which mimics the clinical outcomes seen in UC. This condition results from the interplay between intestinal dysbiosis and a weakened immune response^15^,^16^. Therefore, this research aimed to investigate the anti-ulcerative and antioxidant effects of different concentrations of the hydroalcoholic extract of *A. occidentale* leaves in an *in-vivo* model of colitis induced via acetic acid.

## Methods

### Plant material

The leaves of *A. occidentale* were collected at the Agricultural Research Company of Rio Grande do Norte located in Parnamirim city, RN, Brazil. The leaves were carefully stored in plastic bags and transported to the Pharmacognosy Laboratory of the Department of Pharmacy at the Universidade Federal do Rio Grande do Norte (UFRN) for the preparation of a hydroalcoholic leaf extract. A specimen of the species was deposited in the herbarium of the Department of Botany, Ecology, and Zoology at UFRN for taxonomic identification purposes.

### Preparation and composition of Anacardium occidentale leaf extract

The raw material was dried in an oven with air circulation (temperature ≤ 45°C) and subsequently crushed in a knife mill. Extraction was carried out for seven days by maceration in a mixture of ethanol and water (70:30 v/v) using a ratio of 1:10 (w/v) between the dried leaf material and the solvent solution. The extract was filtered, and the solvent volume was reduced by approximately 70% using a rotary evaporator (IKA, Controle RV 10 CV model, Staufen, Germany) at 40°C. The concentrated solution was then lyophilized to obtain a dry powdered extract^6^. The crude extract was then obtained, and qualitative phytochemical analysis was carried out by thin layer chromatography using pre-coated aluminum sheets with silica gel 60G F254 as adsorbent. Mobile phases with two polarities were used:

- Ethyl acetate:formic acid:acetic acid:water (8:0.25:0.25:1.0, v/v/v/v);
- Toluene:ethyl acetate:formic acid (5:5:0.5, v/v/v).

The chromatograms were visualized using ultraviolet light at 365 nm after spraying with 0.5% natural reagent A (β-aminoethyl diphenylboric acid ester complex). Standards of isoorientin, isoquercetin, quercetin, and kaempferol were used to compare retention factors (Rf) and color spots.

### Animals

This research was conducted in accordance with the guidelines set forth by the National Institutes of Health for the Care and Use of Laboratory Animals, as well as Brazilian Federal Law no. 11,794/2008. The study received approval from the Ethics Committee of the UFNR (protocol no. 002/2014).

Sixty male Wistar rats (250 g, 4 months old from Biotery of Universidade Potiguar) were randomly distributed into six groups (n = 10). They were housed in a biotery with controlled temperature (21 ± 2°C), humidity (60 ± 10%), and luminosity (12:12-h light/dark cycle) with access to food and water *ad libitum*.

### Animal model of colitis induction

Colitis was induced on the third day of treatment with *A. occidentale* hydroalcoholic leaf extract (HEA) through rectal administration of 4% acetic acid (Sigma-Aldrich) in 0.9% saline solution^17^. The animals fasted for 7 hours and were then anesthetized. A sterile pediatric catheter measuring 0.2 mm in diameter was inserted rectally up to 8 cm in the height of the colon. Afterward, 1 mL (4% v/v) of acetic acid (AA) in 0.9% sodium chloride (NaCl) was administered. The animals were kept in the vertical position, and after 30 seconds, the AA was drained.

### Experimental groups

The animals were organized into six experimental groups:

- Control group (C) (n = 10): rats received 0.9% saline solution (orogastric gavage);
- Control colitis (CC) (n = 10): rats received 4% acetic acid in 0.9% saline solution (rectal administration);
- Sz500 colitis (SZC) (n = 10): rats with colitis received sulfasalazine (500 mg/kg of body mass, Apsen Pharmaceuticals, Brazil) diluted in 0.9% saline solution (orogastric gavage);
- HEA50 colitis (HEA50) (n = 10): rats with colitis received the hydroalcoholic extract of *A. occidentale* (50 mg/kg of body mass, orogastric gavage);
- HEA100 colitis (HEA100) (n = 10): rats with colitis received the hydroalcoholic extract of *A. occidentale* (100 mg/kg of body mass, orogastric gavage);
- HEA200 colitis (HEA200) (n = 10): rats with colitis received the hydroalcoholic extract of *A. occidentale* (200 mg/kg of body mass, orogastric gavage).

**Figure 1:**
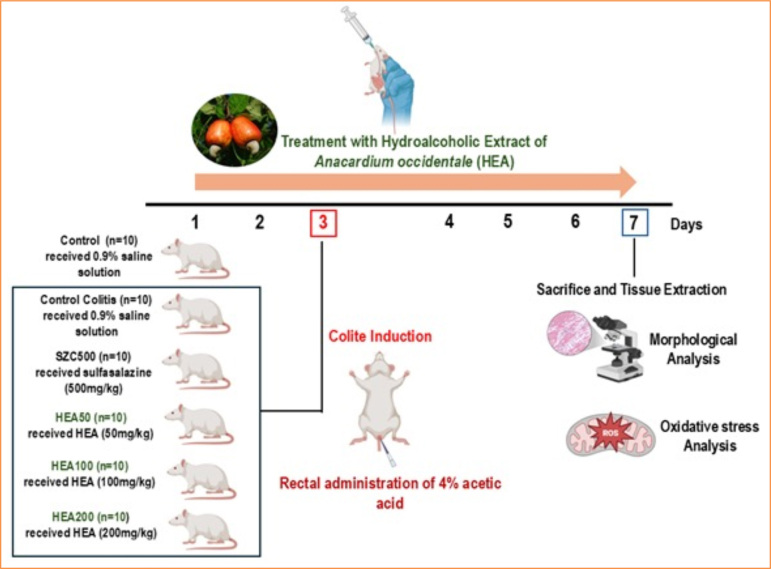
Schematic representation of the experimental design used.

Source: Elaborated by the authors.

Treatments with HEA were administered daily (once a day) for seven consecutive days. Figure 1 details the experimental design covered.

### Food intake and body mass

Daily, food intake was calculated (8 a.m.) by the difference between the food offered and food not consumed after 24 h. Body mass was measured daily (10 h). These procedures were carried out throughout the experimental period.

### Sacrifice and tissue extraction

The animals were fasted (15 hours), deeply anesthetized intraperitoneally (ketamine 300 mg/kg + xylazine 60 mg/kg), and sacrificed by exsanguination. The intestine (colon) was dissected, weighed, and fixed (Millonig formalin) for microscopy techniques or frozen (-80°C) for oxidative stress analysis.

### Morphological analysis

A longitudinal incision of the intestine was made to determine the macroscopic score of colonic lesions, and digital images were captured (Sony, A6400). The criteria below were used to evaluate the degree of injuries and to determine the score according to Wallace’s score^18^ (Table 1).

**Table 1:** Wallace’s score.

Scale	Criteria
0	No damage
1	Hyperemia, without ulcers
2	Linear ulcer without significant inflammation
3	Linear ulcer with inflammation in one site
4	Two or more sites of ulceration/inflammation
5	Two or more sites of ulceration and inflammation or one site of inflammation greater than 1 cm along the length of the colon
6	If the damage covers more than 2 cm along the length of the colon (the scale is increased by one point for each additional centimeter)

Source: Elaborated by the authors.

For histopathological analyses, slides were prepared with colon fragments (5-µm thick) stained with hematoxylin-eosin (Sigma-Aldrich). Fifty random fields per group were photographed and analyzed based on the following histopathological findings: ulceration, hemosiderin, vascular congestion, bleeding, edema, inflammatory infiltrate, and transmural infiltrate, which were classified according to their intensities:

- 0: absent;
- 1: mild (limited involvement of the mucosal layer);
- 2: moderate (involvement of the mucosal and submucosal layers);
- 3: intense (involvement of the mucosa, submucosa and muscular layers)19.

### Oxidative stress analysis

#### Thiobarbituric acid reactive substances

This assay followed the technique proposed in the method described by Draper and Hadley^20^ and was adapted to the colon. In general, damage to membrane lipids was measured by the formation of lipid peroxidation products (malondialdehyde), which are reactive thiobarbituric acid (TBA) heating substances formed during peroxidation in membrane systems and microsomes. Then, 150 μL of the sample was used in 300 μL of 20% trichloroacetic acid (Sigma-Aldrich). The samples were centrifuged for 20 minutes at 2,000 rpm at 4°C, the supernatant was separated into microtubes, and 150 μL of TBA (0.67%) was added. The microtubes were placed in a dry bath (100°C) for 30 minutes, and after cooling, a spectrophotometric reading was performed (532 nm).

#### Protein thiol oxidation

The samples (50 µL) were diluted in 0.1% sodium sulfate, 0.01 M 5,5’-dithiobis (2-nitrobenzoic acid, Sigma Aldrich), and ethanol was added. After 20 minutes, a spectrophotometer was read at 412 nm; the results were shown as µmol/mg protein.

#### Catalase and superoxide dismutase

The enzymatic activity of catalase was obtained by spectrophotometry, according to the proposed technique by Aebi^21^, and adapted to the colon. Then, 20 μL of the sample was used in separate cuvettes (quartz). The samples were incubated in 1,980 μL of buffer solution: 25 mL of phosphate buffer for each 40 μL H_2_O_2_ (0.16%). The concentration of H_2_O_2_ was evaluated for 60 seconds by spectrophotometry (240 nm). Superoxide dismutase (SOD) activity was determined according to the Marklund and Marklund method^22^, and the reading was carried out in a spectrophotometer with the absorbance of 480 nm; results were expressed as U/mg of protein.

#### Statistical analysis

Data were tested for normality (Shapiro-Wilk’s test for a small sample) and homoscedasticity (Bartlett’s test) and presented as mean and standard deviation. Considering *p* < 0.05 as statistically significant, differences between groups were tested by one-way analysis of variance (ANOVA) with Brown Forsythe and Welch test followed by Dunnett T3 post-test (GraphPad Prism v.10.2.1, GraphPad Software, San Diego, CA, United States of America). Five replicates per analysis are the minimum to achieve the probability of occurrence p = (1/2)^5^ = 0.05^23^.

## Results

### Phytochemical analysis

**Figure 2:**
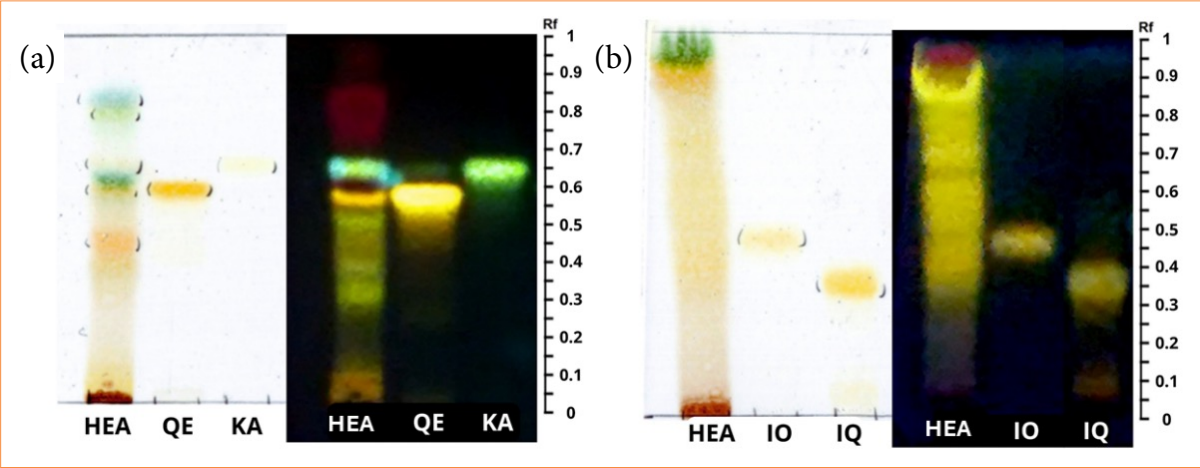
*Anacardium occidentale* extract (HEA) and standards (isoorientin–IO; isoquercetin–IQ; quercetin–QE; and kaempferol–KA) analyzed by thin layer chromatography using aluminum sheets of silica gel F254. Detection: natural reagent A and ultraviolet 365 nm. **(a)** Eluent: ethyl acetate:formic acid:acetic acid:water (8:0.25:0.25:1.0, v/v/v/v). **(b)** Eluent: toluene:ethyl acetate:formic acid (5:5:0.5, v/v/v). Ultraviolet 365 nm.

Source: Elaborated by the authors.

Thin layer chromatography of HEA was revealed with NP-Reagent (specific reagent to detect the presence of flavonoids). When analyzed in visible light and ultraviolet light at 365 nm, HEA revealed yellow and green spots. Using mobile phase 1, in HEA, spots were observed (yellow color at Rf = 0.58 and green color at Rf = 0.64) that matched the commercial standards of quercetin and kaempferol (Fig. 2a; Table 2). In the mobile phase 2, the HEA spots did not show satisfactory resolution. However, when evaluated in ultraviolet light at 365 nm, orange bands were detected that may correspond to the standards of isoquercetin (Rf = 0.46) and isoorientin (Rf = 0.34) (Fig. 2b; Table 2). To summarize the phytochemical analysis, thin layer chromatography results suggested the presence of quercetin, kaempferol, isoquercetin, and isoorientin in the HEA.

**Table 2:** Retention factor (Rf) values and color of thin layer chromatography spots observed in hydroalcoholic extract of *Anacardium occidentale*.

Commercial standard	*Rf* value	Color/ visible light	Color/ultravioleta 365 nm
Quercetin	0.09	Yellow	Yellow
Kaempferol	0.31	Green-yellow	Green
Isoquercetin	0.46	Yellow	Orange
Isoorientin	0.34	Yellow	Orange

Source: Elaborated by the authors.

### Action of Anacardium occidentale extract on body weight, food, and water intake

**Figure 3:**
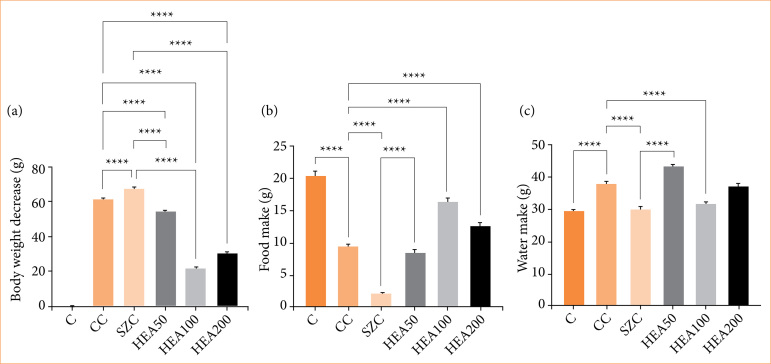
Biometric data of animals. **(a)** Body weight; **(b)** food intake; **(c)** water intake. The data is expressed as mean ± standard deviation. Statistical significance: ****p < 0.0001.

Animals that were induced to colitis (CC) showed significant weight loss. In relation to the CC group, the groups treated with the *A. occidentale* extract presented reduction in body weight loss: HEA50 (-14.3%, *p* < 0.0001); HEA100 (-65.4%, *p* < 0.0001), and HEA200 (-49.1%, *p* < 0.0001). The HEA100 group surpassed the pharmacological effects of sulfasalazine in attenuating body weight loss (-68%, *p* < 0.0001) (Fig. 3a). In parallel, food intake was lower in group CC compared to group C (-53%, *p* < 0.0001). In comparison to the CC group, the HEA100 and HEA200 groups showed recovery in food intake (+70% and +33%; *p* < 0.0001, respectively) (Fig. 3b). Water intake was elevated in the CC group (+28%, *p* < 0.0001) compared to group C and was reduced in the HEA100 and HEA50 groups (-16% and -1%; *p* < 0.0001, respectively) when compared to the CC group (Fig. 3c).

C: control; CC: control colitis; SZC: sulfasalazine (500 mg/kg); HEA50: hydroalcoholic extract of *Anacardium occidentale* (50 mg/kg); HEA100: hydroalcoholic extract of *A. occidentale* (100 mg/kg); HEA200: hydroalcoholic extract of *A. occidentale* (200 mg/kg). Source: Elaborated by the authors.

### Anacardium occidentale extract improves colonic function and lesions caused by induced colitis

**Figure 4:**
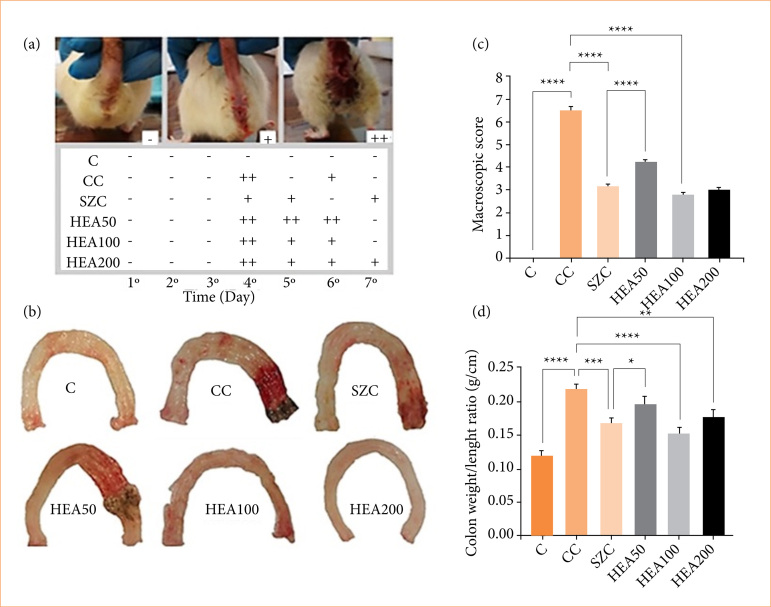
Data relating to the morphophysiological parameters of the animals’ colon. **(a)** Stool evacuation; **(b)** macroscopic analysis of the colon; **(c)** macroscopic lesion score; **(d)** colon weight/length ratio. The data is expressed as mean ± standard deviation. Statistical significance: *p < 0.05; **p < 0.01; ***p < 0.001; ****p < 0.0001.

Stool evacuation was measured over the seven experimental days and was classified as absent (-), moderate (+), and intense (++). As expected, the CC group showed greater fecal elimination (++). Treatments with HEA100 and HEA200 circumvented diarrhea episodes (Fig. 4a). Macroscopic analyses showed extensive areas of lesions in the colon of the CC group. Contrary, the HEA100 and HEA200 groups recovered from these lesions, surpassing the pharmacological effects observed in the sulfasalazine group (Fig. 4b). The macroscopic score of the lesions and of the colon weight/length ratio reinforces these results; when compared to group C, the CC group showed a significant increase in the extent of the lesion areas and in the dimensions of the colon (+650% and +75% *p* < 0.0001). In relation to the CC group, the HEA100 group showed significant reduction in the extent of lesion areas with recovery in colon dimensions (-47% and -28%, *p* < 0.0001) (Figs. 4c and 4d).

C: control; CC: control colitis; SZC: sulfasalazine (500 mg/kg); HEA50: hydroalcoholic extract of *Anacardium occidentale* (50 mg/kg); HEA100: hydroalcoholic extract of *A. occidentale* (100 mg/kg); HEA200: hydroalcoholic extract of *A. occidentale* (200 mg/kg). Source: Elaborated by the authors.

### Anacardium occidentale extract recovers tissue damage in the intestinal wall caused by induced colitis

**Figure 5:**
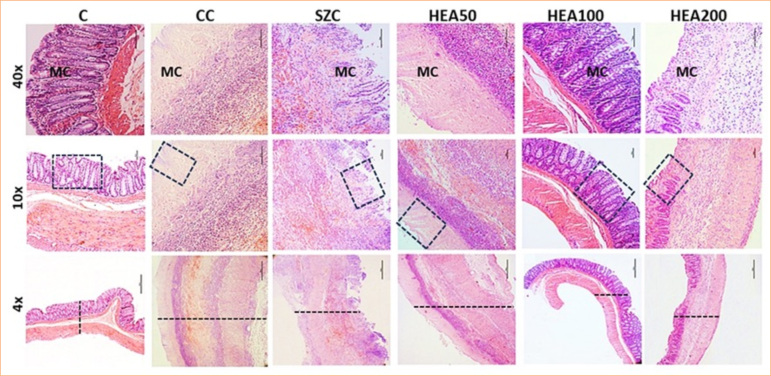
Effects of hydroalcoholic extract of *Anacardium occidentale* (HEA) on colon histopathology of rats with colitis. 4x magnification: changes in the intestinal wall thickness (dashed line). 10x magnification: detail of changes in the colon mucosa (dashed rectangle). 40x magnification: mucosa (MC) highlighting changes in the villi. Treatment with HEA100 decreased the thickness of the intestinal wall and recovered the mucosa and the distribution pattern of the villi. Hematoxylin-eosin.

**Figure 6:**
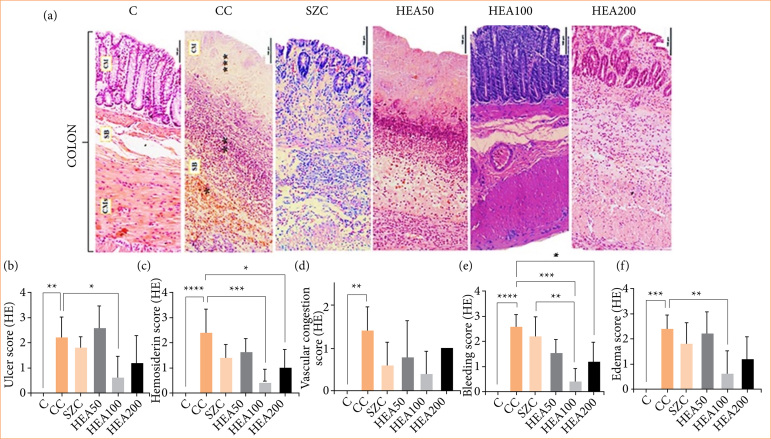
**(a)** Representative photomicrographs of the intestinal colon. The CC group showed destruction of the mucosal layer (***), presence of transmural infiltrate and bleeding (*), inflammatory infiltrate, and edema (**). Microscopic score of **(b)** ulcer, **(c)** hemosiderin, **(d)** vascular congestion, **(e)** bleeding, and **(f)** edema. Treatment with HEA100 recovered the histopathological changes caused by the induced colitis. Magnification: 40x. Hematoxylin-eosin. The data is expressed as mean ± standard deviation. Statistical significance: *p < 0.05; **p < 0.01; ***p < 0.001; ****p < 0.0001.

Histopathological analyses showed severe changes induced by AA in the colon of animals in the CC group, characterized by thickening of the colon wall with significant reduction of villi in the mucous layer (necrosis), presence of inflammatory infiltrate and edema in the submucosal layer, and transmural bleeding (Figs. 5 and 6). Treatment with HEA100 attenuated the severity of cellular damage in the colon caused by the local action of acetic acid, surpassing the pharmacological effects observed in the sulfasalazine group and equaling the typical histological pattern of the colon observed in group C (Figs. 5 and 6). The histopathological score corroborates these findings; when compared with group C, group CC showed a significant increase in the degree of ulcer (+220%, *p* < 0.01), hemosiderin (+240%, *p* < 0.0001), vascular congestion (+140%, *p* < 0.01), bleeding (+260%, *p* < 0.0001) and edema (+240, *p* < 0.001). Treatment with HEA100 attenuated the score of these parameters: ulcer -73%, *p* < 0.05; hemosiderin -83%, *p* < 0.001; vascular congestion -71%; bleeding -85%; *p* < 0.001; and edema -75%; *p* < 0.01 (Fig. 6b and 6f).

C: control; CC: control colitis; SZC: sulfasalazine (500 mg/kg); HEA50: hydroalcoholic extract of *Anacardium occidentale* (50 mg/kg); HEA100: hydroalcoholic extract of *A. occidentale* (100 mg/kg); HEA200: hydroalcoholic extract of *A. occidentale* (200 mg/kg). Source: Elaborated by the authors.

C: control; CC: control colitis; SZC: sulfasalazine (500 mg/kg); HEA50: hydroalcoholic extract of *Anacardium occidentale* (50 mg/kg); HEA100: hydroalcoholic extract of *A. occidentale* (100 mg/kg); HEA200: hydroalcoholic extract of *A. occidentale* (200 mg/kg); CM: mucous layer; CMs: muscular layer; SB: submucosa. Source: Elaborated by the authors.

### Anacardium occidentale extract mitigated oxidative stress

**Figure 7:**
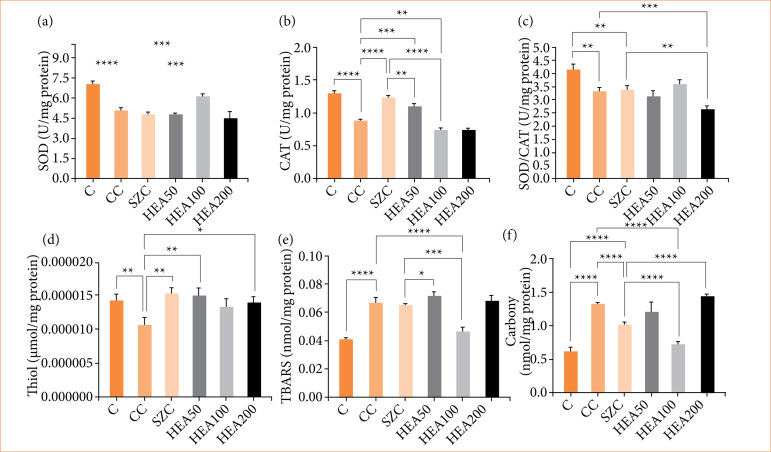
Action of hydroalcoholic extract of Anacardium occidentale on markers of oxidative stress. **(a)** Superoxide dismutase (SOD), **(b)** catalase, **(c)** SOD/CAT, **(d)** thiol, € thiobarbituric acid reactive substances (TBARS), and **(f)** carbonyl. The data is expressed as mean ± standard deviation. Statistical significance: *p < 0.05; **p < 0.01; ***p < 0.001; ****p < 0.0001.

The activity of the antioxidant enzymes SOD, CAT, and thiol was significantly reduced in the colons of animals in group CC compared to group C (-27%, -31%, and -25%; *p* < 0.0001, respectively). The joint action of SOD/CAT also decreased in group CC when compared to group C (-24%, *p* < 0.001). Treatments with HEA100 and HEA50 recovered the antioxidant activity of SOD (+20%, *p* < 0.001), CAT (+23%, *p* < 0.001), and thiol (+24% and +41%, *p* < 0.01, respectively) (Figs. 7a and 7d). The levels of lipid peroxidation (TBARS) and protein oxidation (carbonyl) were increased in the colons of animals in group CC when compared to group C (+67% and +106%; *p* < 0.0001, respectively). Treatment with HEA100 improved the response of these two oxidative stress enzymes (-31% and -45%; *p* < 0.0001, respectively) (Figs. 7e and 7f).

C: control; CC: control colitis; SZC: sulfasalazine (500 mg/kg); HEA50: hydroalcoholic extract of *Anacardium occidentale* (50 mg/kg); HEA100: hydroalcoholic extract of *A. occidentale* (100 mg/kg); HEA200: hydroalcoholic extract of *A. occidentale* (200 mg/kg). Source: Elaborated by the authors.

## Discussion

In this research, we examined the effects of the HEA in a model of colitis induced by AA. The AA-induced colitis serves as an experimental model for inflammatory bowel disease, resembling the pathophysiology of human UC. This condition is characterized by lesions in the intestinal mucosa, inflammation in the submucosa, diarrhea, and weight loss^24^,^25^. UC can be triggered by various factors, including lifestyle choices (such as diet and physical activity) and exposure to antibiotics. These factors can influence the composition of the intestinal microbiota, affect the production of microbial metabolites, and alter the immune response of the intestinal mucosa^16^.

According to these findings and previous studies^26^,^27^, our research indicates that animals in the colitis group (CC) experienced reduction in body weight, increased stool elimination, and higher water intake. They also exhibited severe damage to the colon mucosa, including loss of villi (necrosis), ulceration, inflammatory infiltration, and heightened oxidative stress. Our analysis revealed that treatment with a hydroalcoholic extract of *A. occidentale* leaves, at the concentration of 100 mg/kg of body mass, for one week was sufficient to reverse these changes induced by colitis. This treatment demonstrated significant antiulcerative and antioxidant potential.

Colitis causes weight loss due to dietary deficiencies generated by decreased appetite, malabsorption, loss of body fluids, and diarrhea^28^,^29^, effects that occur with greater water intake, all observed in the CC group. Macroscopic examination showed extensive areas of lesions in the colon of animals in the CC group; in parallel, greater colon weight/length was observed in these animals. Histopathological analyses showed an increase in the thickness of the colon wall caused by a considerable reduction in villi, an increase in ulcer scores, hemosiderin, vascular congestion, bleeding, and edema. The daily action of HEA100 for seven days improved diarrhea, increased food intake, attenuated weight loss, and recovered the macroscopic and histological parameters of the colon, restoring the intestinal morphophysiology of these animals.

As a result of the tissue changes observed in the CC group, there was an imbalance in the oxidative homeostasis of the colon. This included reduction in the activity of the antioxidant system, specifically SOD, CAT, and thiol, alongside increased levels of lipid peroxidation (measured by TBARS) and protein oxidation (indicated by carbonyl). The worsening of colitis is closely linked to deficiencies in antioxidant enzymes and heightened oxidative stress^30^,^31^. Our findings indicate that treatments with HEA50 and HEA100 enhanced the activity of SOD, CAT, and thiol, while also decreasing the levels of TBARS and carbonyl, thereby contributing to the restoration of the antioxidant system’s homeostasis.

Our data indicated that the concentration of 100 mg/kg of body mass of HEA exhibited the most significant antiulcerative and antioxidant therapeutic effects in the induced colitis model. Concentrations of 50 mg/kg appeared to have sub-therapeutic effects, while 200 mg/kg showed toxic effects. Interestingly, our findings revealed that the therapeutic effects of HEA100 surpassed those of sulfasalazine, which is a standard drug used for the treatment and clinical management of UC^32^,^33^.

In alignment with the therapeutic properties of the hydroalcoholic extract of *A. occidentale* leaves demonstrated in this study, phytochemical analysis indicated that HEA is abundant in flavonoids, particularly quercetin, isoorientin, isoquercetin, and kaempferol.

Flavonoids exhibit powerful anti-inflammatory and antioxidant properties, positively impacting in numerous biological processes^34^,^35^. Research indicates that flavonoids improve UC through their anti-inflammatory and antioxidant effects by inhibiting the NF-kB signaling pathway^36^. Quercetin has been found to play a significant role in preventing gastric mucosal injuries across various experimental models. It enhances the expression of neutral glycoproteins, reduces the levels of pro-inflammatory cytokines, and diminishes lipid peroxidation^37^. Isoorientin, on the other hand, has shown to alleviate damage in the intestines of animals with UC by increasing the levels of total SOD and glutathione peroxidase, while simultaneously decreasing the levels of malondialdehyde and hydrogen peroxide (H_2_O_2_)^38^.

**Figure 8:**
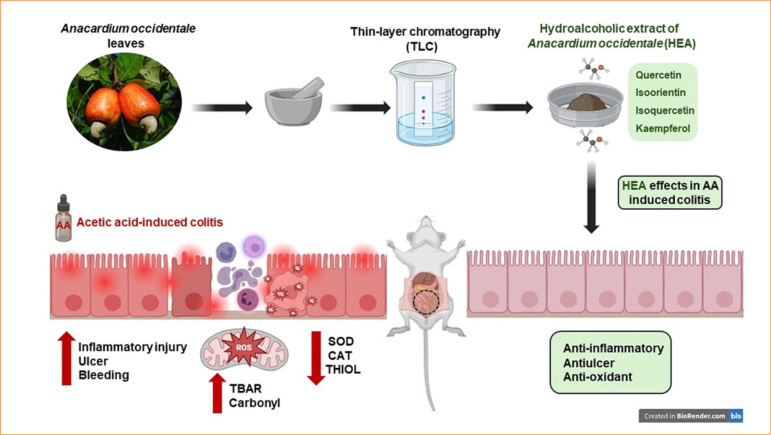
Summary of the main findings. The acetic acid (AA)-induced colitis model demonstrated several morphofunctional changes in the colon, including inflammation, ulcers, bleeding, and increased oxidative stress. The hydroalcoholic extract of *Anacardium occidentale* leaves (HEA), which is rich in flavonoids such as quercetin, isoorientin, isoquercetin, and kaempferol, helped mitigate these changes. It displayed antiulcer, antioxidant, and anti-inflammatory effects, highlighting its potential as a promising therapeutic agent for the treatment of colitis.

SOD: superoxide dismutase; CAT: catalase; TBAR: thiobarbituric acid reactive substance. Source: Elaborated by the authors.

This data supports our findings and suggests that HEA is a potent therapeutic agent for colitis treatment with antiulcerative, antioxidant, and anti-inflammatory properties. Figure 8 summarizes the key results presented in this research.

## Conclusion

The hydroalcoholic extract of A. occidentale leaves mitigated intestinal changes in the experimental model of colitis induced by AA. Treatment with HEA100 (100 mg/kg of body mass) surpassed the pharmacological effects of sulfasalazine, recovered severe damage to the colon mucosa, and positively modulated oxidative stress markers, proving to be a promising therapeutic agent for treating colitis. The concentration of 50 mg/kg of HEA exerted subtherapeutic effects, while the concentration of 200 mg/kg seemed to exceed its bioavailability capacity, resulting in the absence of its therapeutic effect with a possible toxic effect.

## Data Availability

The data will be available upon request.
